# Reimagining Oncologic Drugs in Atherosclerosis: Emerging Mechanisms and Therapeutic Potential

**DOI:** 10.3390/biomedicines13092282

**Published:** 2025-09-17

**Authors:** George Liu, Guillaume De Vlaminck, Osayamen Atekha, Eric P. Grewal, Rishab Ramapriyan, Gautam Agarwal

**Affiliations:** 1 Medical College of Georgia, Augusta University, Augusta, GA 30912, USA; 2 Department of Neurosurgery, Massachusetts General Hospital, Boston, MA 02114, USA; 3 Harvard Medical School, Boston, MA 02115, USA; 4 Department of Neurosurgery, University of Pennsylvania, 3400 Civic Center Boulevard, Philadelphia, PA 19104, USA; 5 Department of Vascular Surgery, Medical College of Georgia at Augusta University, Augusta, GA 30912, USA

**Keywords:** atherosclerosis, oncology, angiogenesis, immunotherapy, targeted therapy

## Abstract

Atherosclerosis is a chronic vascular disease that underlies the pathogenesis of both peripheral arterial disease and coronary artery disease, two of the leading causes of morbidity and mortality worldwide. Characterized by the accumulation of lipids, chronic inflammation, and fibrotic remodeling within vasculature, atherosclerosis involves a complex interplay of endothelial dysfunction, immune dysregulation, vascular smooth muscle cell proliferation, and maladaptive neovascularization. Increasing evidence now suggests that atherosclerosis has notable overlap with cancer biology, including sustained proliferative signaling, evasion of immune surveillance, angiogenesis, and resistance to cell death. These shared molecular features have prompted growing interest in the potential repurposing of oncologic treatments in the modulation of atherosclerotic disease. While preclinical data are promising, successful translation and integration of oncologic therapeutics will require overcoming critical barriers, including drug toxicity, long-term safety, regulatory constraints, and cost-effectiveness. Future work should focus on biomarker-guided patient selection, dose optimization, and targeted delivery systems to minimize off-target effects while enhancing efficacy.

## 1. Introduction

Atherosclerosis remains a leading cause of morbidity and mortality worldwide, driven by complex interactions between lipids, the immune system, and the endothelium that ultimately thicken arteries and can result in thrombosis and/or infarction [1,2]. The initial understanding of atherosclerotic plaque formation centered around the idea that it was primarily the result of excess lipids [3] causing fatty streaks along the intimal layer of arteries. While lipid deposition remains a central feature of the disease, atherosclerosis is now recognized as a multifactorial process combining innate immune activation, chronic inflammation, and tissue remodeling [4].

Current evidence suggests that endothelial injury, triggered by risk factors such as cigarette smoking and hypertension, helps initiate plaque development, enabling subendothelial accumulation and oxidation of low-density lipoprotein (LDL) [5]. Within plaques, an oxidative and pro-adhesive state drives recruitment of circulating monocytes and differentiation into macrophages, which engulf the modified LDL and become foam cells [6,7]. These foam cells, in turn, release pro-inflammatory cytokines that amplify immune cell recruitment and propagate chronic inflammation, namely the proliferation and activation of vascular smooth muscle cells that drive plaque progression [8]. Thus, inflammation is not merely a consequence, but a driving force present throughout all stages of plaque initiation, progression, and eventual destabilization [4,9].

Strikingly, the mechanisms of atherogenesis closely mirror the biological hallmarks of cancer, with cells within atherosclerotic plaques acquiring many characteristics and signaling states similar to those within tumors [10,11]. One important overlapping characteristic is dysregulated cell proliferation, which can induce oxidative stress, a driver of both atherosclerotic plaques and various types of cancer [12,13]. Additional shared factors include alterations in cell adhesion molecules, dysregulated protease expression, and angiogenesis [14]. This convergence of mechanisms raises the question of whether therapeutic strategies developed in the field of oncology may also have efficacy in modulating atherosclerotic disease.

In this review, we will first examine the molecular intersections between atherosclerosis and cancer. We will then explore classes of oncologic drugs that are currently being investigated for atherosclerotic disease and those that hold theoretical potential for atherosclerotic treatment along with their associated toxicities. Finally, we will discuss translational implications, the current limitations, and future directions of integrating oncology-based therapeutics into the management of vascular disease.

Methodologically, this was a scoping review created by a targeted literature search in PubMed and Embase. Articles were included based on relevance to pathophysiology of atherosclerosis and oncology, prioritizing studies with novel mechanistic insights, clinical outcomes data, or broad impact on the field.

## 2. Pathophysiologic Parallels Between Atherosclerosis and Cancer

### 2.1. Aberrant Cell Proliferation and Warburg-like Metabolism

A core similarity between atherosclerosis and cancer is the presence of aberrant and sustained cellular proliferation. In atherosclerosis, vascular smooth muscle cells (VSMCs) respond to atherogenic inflammation by switching from a contractile to a synthetic phenotype [15]. This phenotypic modulation is accompanied by increased proliferation, migration into the intima, and secretion of extracellular matrix components such as collagen and fibronectin, leading to plaque expansion and vascular remodeling [16]. Such proliferative behavior bears similarity to that of neoplastic cells, which similarly escape normal growth control mechanisms to drive tumor formation [17].

At the molecular level, the platelet-derived growth factor (PDGF), transforming growth factor-beta (TGF-β), and phosphoinositide 3-kinase/protein kinase B/mammalian target of rapamycin (PI3K/AKT/mTOR) axis are heavily implicated in both cancer biology and atherogenesis [18]. Activation of the PI3K/AKT/mTOR cascade in VSMCs promotes protein synthesis, prevents apoptosis, and contributes to plaque thickening; this is mirrored by the role of mTORC1 in promoting cancer cell growth by enhancing glycolysis and lipid synthesis [19,20].

The PI3K/AKT/mTOR pathway is pleiotropic, acting as a central effector in multiple core cellular functions, ranging from metabolism to autophagy inhibition and resistance to cell death. In both cancer and atherosclerosis, this axis facilitates survival in nutrient- or oxygen-deprived environments, such as within the core of tumor growth or deep within atherosclerotic plaques [21].

Relatedly, TGF-β can be tumor-suppressive in early cancer but is pro-fibrotic and oncogenic in later stages [22]. Within a vascular scope, TGF-β promotes EndoMT (endothelial-to-mesenchymal transition) and Smad3-mediated transcription, processes that both may cooperate with PI3K/AKT signaling to drive synthetic VSMC transformation [23,24].

Building on these molecular similarities, metabolic reprogramming provides a further point of convergence. The Warburg effect, originally described in cancer cells, refers to the preferential conversion of glucose to lactate, otherwise known as aerobic glycolysis, even in the presence of sufficient oxygen [25]. This metabolic shift supports rapid ATP generation and provides biosynthetic intermediates for nucleotide, amino acid, and lipid synthesis, key functions required for cancer cells to meet the high metabolic demands of unchecked proliferation [26,27]. Recent studies have demonstrated that similar metabolic shifts occur in atherosclerotic lesions [28]. Activated macrophages, endothelial cells, and VSMCs within plaques exhibit upregulated glycolytic enzymes such as hexokinase 2 (HK2) and lactate dehydrogenase A (LDHA), alongside increased glucose uptake through GLUT1 [29]. These enhanced glycolytic shifts support cellular proliferation and inflammatory signaling, contributing to plaque development and instability [29]. The consequence of this metabolic adaptation is multifaceted: it not only sustains cellular growth, but also promotes extracellular acidification and matrix degradation, which may weaken plaque structure and contribute to instability [30].

Moreover, the transcription factors hypoxia-inducible factor 1a (HIF-1α) and c-Myc, known drivers of the Warburg phenotype in tumors, are also upregulated in atherosclerotic tissue, supporting glycolysis, angiogenesis, and inflammatory gene expression within developing plaques [10,31,32,33]. This overlap suggests that targeting glycolytic metabolism, an ongoing therapeutic strategy in oncologic treatments, may also hold promise for modulating vascular inflammation and proliferation in atherosclerosis.

### 2.2. Chronic Inflammation and Immune Evasion

Chronic inflammation, once viewed as a consequence of atherosclerosis, is now recognized as a driver of both plaque development and destabilization [9]. Similarly, in cancer, pathogenic inflammation plays a dual role, enabling genomic instability to initiate cancer while also creating a hostile tumor microenvironment to hamper antitumor immunity [34]. This chronic inflammatory state forms a critical point of intersection between the two diseases.

JAK/STAT and MAPK are two key pathways that are activated in vascular inflammation and tumor biology [35]. Intrinsically, inflammatory cytokines such as interleukin-6 (IL-6) and interferon-gamma (IFN-γ) may be released by immune cells within plaques and activate JAK/STAT signaling; while pro-inflammatory immune cells, mediators within this cascade (e.g., STAT3), also specifically sustain vascular smooth muscle cell (VSMC) proliferation [10,36]. Extrinsically, cytokines locally promote atherosclerosis by amplifying the secretion of chemokines and upregulating endothelial adhesion molecules, such as ICAM-1 and VCAM-1, which ultimately facilitate monocyte extravasation and foam cell formation [36]. In the context of cancer, cytokines like IFN-γ may locally promote tumor regression by activating antitumoral immune cells in the tumor microenvironment but can also contribute to the systemic immune dysregulation associated with cancer when secreted systemically [37]. Likewise, MAPK pathways, especially key components ERK1/2 and p38, are activated by oxidized lipids and mechanical stress in the vasculature, promoting monocyte recruitment and endothelial dysfunction [38]. These same cascades are frequently disrupted and hijacked in cancer to foster unchecked proliferation, immune resistance, and neoangiogenesis [10,39]. Overall, these pathways can operate in tandem, promoting inflammatory amplification loops that blur the distinction between vascular lesion and tumor biology.

In addition to pathways responsible for inflammation is the emerging recognition that immune checkpoint mechanisms are also active within atherosclerotic plaques. The programmed death-1 (PD-1) receptor and its ligand PD-L1, well-known for suppressing T-cell activity in tumors, have been detected within vascular tissue [40]. Their upregulation in atherosclerosis appears to blunt T-cell responses and reduce inflammation [41]. Within atherosclerosis, there is evidence suggesting that the PD-1/PD-L1 pathway is important in downregulating proatherogenic T-cell responses, promoting immune tolerance, and upregulating antiatherogenic mechanisms [42]. Similarly, cytotoxic T-lymphocyte-associated antigen 4 (CTLA-4), another immune checkpoint molecule, is expressed within plaques and modulates local immune responses [43]. In the context of cancer, PD-1/PD-L1 and CTLA-4 are inhibited with monoclonal antibodies to enhance antitumor T cells immunity, raising concerns about the potential unintended cardiovascular and peripheral vascular consequences of immune checkpoint inhibitors. By disrupting immune regulation, these agents may inadvertently trigger vascular inflammation or plaque destabilization.

### 2.3. Neovascularization and Angiogenesis

As both atherosclerotic plaques and tumors grow, they often outpace their existing blood supply, leading to hypoxia and the subsequent reliance on angiogenesis [44]. Angiogenesis, which is the formation of new blood vessels from pre-existing vasculature, is a hallmark feature of both cancer and advanced atherosclerosis; in both cases, it serves to sustain the lesion by restoring oxygen and nutrient delivery, but the resulting vasculature is frequently abnormal and pathologic [45,46].

Vascular endothelial growth factor A (VEGF-A) plays a central role within both disease processes. In response to hypoxia or intimal damage, VEGF-A expression is markedly increased in both tumors and atherosclerotic lesions, triggering endothelial cell proliferation and new vessel formation [47,48]. In atherosclerosis, hypoxic conditions arise due to reduced perfusion within the subendothelial compartment and increase as plaques thicken [47]. In turn, VEGF expression is upregulated in cardiac myocytes, vascular smooth muscle cells, and macrophages, facilitating the growth of intraplaque neovessels [46,49].

However, these neovessels are immature, disorganized, and fragile. Lacking stabilizing pericytes and smooth muscle support, they are highly prone to leakage and rupture, leading to intraplaque hemorrhage, thrombus formation, and plaque destabilization [50]. These characteristics are shared with the fragile and leaky vasculature seen in tumors, which results in suboptimal flow and further hypoxia, leading to a recurrent cycle of VEGF production and metastatic potential [51,52]. Hence, both disease processes have the potential of becoming systemic and diffuse.

Therapeutic strategies that target VEGF or its downstream effectors have shown efficacy in normalizing tumor vasculature and limiting progression. Given the shared angiogenic mechanisms in cancer and atherosclerosis, there is a growing rationale for applying anti-angiogenic agents in the context of vascular disease as well [53]. These treatments could potentially stabilize vulnerable plaques and reduce vascular events, especially in high-risk patients.

### 2.4. Resistance to Apoptosis

Finally, pathologic cells in atherosclerosis and cancer have been well-documented in their ability to evade programmed cell death [54]. In cancer, resistance to apoptosis enables malignant cells to survive under hostile conditions, including chemotherapy and immune surveillance [55]. Similarly, within atherosclerotic plaques, vascular smooth muscle cells and macrophages often resist apoptosis, allowing them to persist and contribute to chronic inflammation and lesion growth [2]. This resistance is mediated by the upregulation of anti-apoptotic proteins such as BCL-2 and survivin, which are also commonly expressed in cancer [56]. These proteins disrupt intrinsic apoptotic signaling pathways, preventing mitochondrial cytochrome c release and caspase activation [57]. As a result, VSMCs and macrophages are shielded from oxidative stress and inflammatory mediators that would otherwise induce cell death.

In addition to molecular regulators, intracellular signaling pathways such as PI3K and nuclear factor kappa-light-chain-enhancer of activated B cells (NF-κB) play critical roles in promoting vascular smooth muscle cell (VSMC) survival. Studies have demonstrated that survivin overexpression and PI3K/NF-κB activation in VSMCs contribute to apoptosis resistance, mirroring survival mechanisms seen in cancer cells [58,59]. NF-κB functions as both a pro-inflammatory and anti-apoptotic transcription factor by promoting cytokine expression while concurrently inhibiting cell death through gene regulation [60].

This convergence of pro-survival pathways highlights a potential therapeutic target in atherosclerosis. Inhibitors of BCL-2 and NF-κB, already approved or in development for oncologic use, may be repurposed to modulate plaque composition and stability by selectively inducing apoptosis in pathological vascular cells.

## 3. Oncologic Drug Classes with Potential Anti-Atherosclerotic Applications

### 3.1. Tyrosine Kinase Inhibitors (TKIs)

Drugs like imatinib, sorafenib, and dasatinib inhibit receptors such as PDGFR and VEGFR, which are involved in VSMC proliferation and angiogenesis. Preclinical models suggest TKIs may reduce neointimal hyperplasia and vascular remodeling.

Tyrosine kinase inhibitors (TKIs) are small molecules that prevent tyrosine kinases from phosphorylating their substrates, thereby altering downstream intracellular signaling pathways [61]. In cancer cells, tyrosine kinases are often constitutively activated or inactivated, leading to dysregulation of key cellular processes such as proliferation, differentiation, migration, apoptosis, and cell survival. The introduction of TKIs into oncology has enabled more targeted treatment approaches, offering improved specificity and safety profiles compared to conventional chemotherapy. Currently, TKIs are used in the treatment of various malignancies, each with distinct kinase targets. Since kinases also play crucial roles in vascular, metabolic, and myocardial regulation, the use of TKIs has a heterogeneous impact on the cardiovascular system [62].

In the following section, we explore how TKIs exert effects ranging from vascular toxicity and pro-atherogenicity to anti-atherogenic and lipid-lowering properties, highlighting their potential for repurposing in atherosclerotic cardiovascular disease.

#### 3.1.1. BCR-Abl Inhibitors

BCR-Abl-targeting TKIs inhibit the activity of the BCR-Abl fusion protein, which is characteristic of chronic myeloid leukemia (CML) and Philadelphia chromosome-positive acute lymphoblastic leukemia (Ph+ ALL) [63].

Imatinib, the first-generation BCR-Abl inhibitor, remains the gold standard in CML treatment. Beyond its oncologic potential, imatinib has also been shown to have positive effects on vascular disease in animal models [64]. In cholesterol-fed rabbits, imatinib reduced atherosclerosis progression and endothelial dysfunction, potentially through decreased serum cholesterol, inflammatory cell infiltration, and oxidative enzyme levels [65]. In a pro-atherogenic mouse model, imatinib also lowered plasma cholesterol and increased plaque stability [66]. Conversely, nilotinib (second-generation) and ponatinib (third-generation) have been associated with increased cardiovascular risk, primarily due to pro-thrombotic states linked to upregulated coagulation factors [66,67]. Notably, imatinib has been shown to reduce blood glucose levels, an effect not seen with nilotinib, which may explain part of its atheroprotective profile [64,68]. Imatinib may also attenuate diabetes-associated atherosclerosis, as evidenced in a murine model [69].

CML patients treated with nilotinib or ponatinib have a higher incidence of vascular events compared to those receiving imatinib. For nilotinib, reported complications include coronary artery disease, acute coronary syndrome, myocardial infarction, cerebrovascular events, peripheral arterial occlusive disease (PAOD), and arrhythmias [64,70]. Arterial occlusive disease may be driven by nilotinib’s direct pro-atherogenic and anti-angiogenic effects on vascular endothelial cells [71]. Ponatinib, often used in patients resistant to first- and second-generation TKIs, has been associated with a significantly elevated risk of cardiac, cerebrovascular, and peripheral arterial events [72,73,74]. However, data on ponatinib’s direct vascular effects remain limited [64].

Another BCR-Abl-targeting TKI, sasatinib, also appears to be linked to lower frequencies of adverse cardiovascular events, with the exception of pulmonary hypertension [75,76]. In a retrospective study of CML/ALL patients treated with dasatinib (median duration: 19 months), no correlation was found with symptomatic PAD [77]. In contrast to the pro-atherogenic effects observed with nilotinib, dasatinib has shown atheroprotective properties in murine models. In hypercholesterolemic mice, dasatinib reduced atherosclerotic lesion formation, likely via decreased oxidized LDL uptake by macrophages [78]. Dasatinib may also promote macrophage polarization toward an anti-inflammatory phenotype and reduce vascular infiltration, suggesting an immunomodulatory mechanism [79]. In another study, dasatinib combined with quercetin (as a senolytic regimen) improved vascular function in atherosclerotic mice, potentially offering cardiovascular benefits [80].

#### 3.1.2. Src Kinase Inhibitors

Src family kinases are involved in various cellular processes, including proliferation, adhesion, migration, inflammation, and vascular permeability [81]. Saracatinib, a non-selective TKI targeting Src kinases (and with some affinity for BCR-Abl), is currently under investigation in clinical trials for cancers such as breast and lung cancer [82].

Saracatinib has been proposed as an adjunct to lipid-lowering therapy in atherosclerotic cardiovascular disease (ASCVD), owing to its anti-inflammatory properties [82]. It exerts effects not only on circulating immune cells, but also directly on human atherosclerotic plaques, where it reduces plaque burden and inflammation, outperforming atorvastatin in mouse models [82]. Additionally, saracatinib has shown efficacy in preventing intermittent hypoxia-induced atherosclerosis in OSAS mouse models and in reducing LDL and monocyte transendothelial migration in human endothelial cells [83]. Given the central role of inflammation and hypoxia in ASCVD pathophysiology, repurposing such TKIs as targeted therapies for atherosclerosis may hold promise, particularly for high-risk disease or to prevent severe plaques from progressing.

#### 3.1.3. VEGF Signaling Pathway Inhibitors (VSPIs)

Sorafenib, a multi-target TKI used in advanced renal cell carcinoma (RCC) and hepatocellular carcinoma (HCC), inhibits both tumor proliferation and angiogenesis by targeting the VEGF signaling pathway among others [84,85].

In a phase III trial in RCC, 4.9% of sorafenib-treated patients experienced cardiac ischemia or infarction, compared to 0.4% in the placebo group [85]. Similar trends were observed in an HCC trial (3% vs. 1%) [86]. Although these outcomes suggest potential vascular toxicity, confounding due to longer treatment duration in the sorafenib group cannot be excluded [85]. A meta-analysis further demonstrated that sorafenib increases the risk of hypertension, a known risk factor for atherosclerosis [87]. Mechanistically, VEGF inhibition may reduce nitric oxide availability, increasing peripheral vascular resistance. While the link with hypertension is well established, additional research is needed to clarify the broader cardiovascular impact of VEGF inhibitors like sorafenib. Importantly, similar to BCR-Abl inhibitors, several VEGF-targeting TKIs cause QTc interval prolongation. This class effect may modestly increase the risk of sudden cardiac death in susceptible individuals [87].

In conclusion, the cardiovascular effects of TKIs are heterogeneous. Among TKIs used in cancer, imatinib and dasatinib show consistent anti-atherogenic and anti-inflammatory effects, supporting their consideration for therapeutic repurposing. Saracatinib, though not yet approved, demonstrates promising vascular benefits due to its immunomodulatory action.

### 3.2. Immune Checkpoint Inhibitors (ICIs)

PD-1 and CTLA-4 inhibitors restore T-cell function but have paradoxical effects in cardiovascular disease. While some data suggest immune reactivation may promote plaque regression, others caution against immune-related adverse events, including accelerated atherosclerosis. Thus, a delicate balance may be required when attempting to modulate this axis with immune checkpoint agonists.

Together with targeted therapies, immune checkpoint inhibitors (ICIs) have revolutionized the treatment landscape for a broad range of solid tumors and hematologic malignancies. Their mechanism of action involves releasing inhibitory brakes on T cells, thereby activating the immune system and eliciting an anti-tumor immune response [88]. As of January 2024, a total of 11 ICIs have received approval for more than 40 distinct indications across various cancer types, reflecting the growing diversity of checkpoint targets and cancer types under treatment [89]. The most commonly used ICIs are monoclonal antibodies targeting cytotoxic T-lymphocyte-associated antigen 4 (CTLA-4), programmed cell death protein 1 (PD-1), and its ligands PD-L1 and PD-L2. Over time, anti-PD-1 and anti-PD-L1 antibodies have demonstrated superior clinical efficacy and tolerability compared to CTLA-4 inhibitors, which has contributed to their broader clinical adoption [88].

Despite their transformative impact, ICIs are associated with a wide spectrum of immune-related adverse events (irAEs), which occur in approximately 70 to 90 percent of treated patients and may affect virtually any organ system due to systemic immune activation [90]. Cardiovascular irAEs, although less common, are increasingly recognized. These include myocarditis, which occurs in 0.5 to 1.7 percent of ICI-treated patients and is associated with a mortality rate of 37.7 percent, significantly higher than that of non-ICI myocarditis [91,92]. Although left ventricular dysfunction is observed in fewer than 50 percent of these cases, arrhythmias and heart failure are frequent complications [93,94]. Pericardial disease, including pericarditis and pericardial effusion, is also reported and carries a high case fatality rate [91,95].

Additional cardiovascular toxicities include vasculitis, atherosclerosis-related events such as acute coronary syndrome (ACS), and venous thromboembolic events [91,96,97,98]. Non-myocarditic left ventricular dysfunction, takotsubo syndrome, and arrhythmias are also known adverse effects [99]. Moreover, ICIs have been associated with QTc interval prolongation [87].

A growing body of evidence suggests that ICIs may accelerate atherosclerosis and increase the risk of ACS through changes in plaque composition [100]. A matched cohort study revealed a threefold increase in atherosclerosis-related cardiovascular events among ICI-treated patients [96]. A follow-up imaging analysis on a subset of these patients, using a case-crossover design, demonstrated a more than threefold acceleration in total aortic plaque progression.

Mechanistically, inhibition of the PD-1 and CTLA-4 pathways increases infiltration of CD4+ and CD8+ T cells and macrophages into atherosclerotic plaques, promotes apoptosis, and leads to a fourfold expansion of the necrotic core, resulting in increased plaque vulnerability [43,101].

However, the cardiovascular effects of ICIs appear to be highly dependent on the specific immune checkpoint target. Magrolimab, a monoclonal antibody targeting the phagocytic checkpoint CD47 on macrophages, has, conversely, shown potential anti-atherosclerotic properties. CD47, a negative regulator of the macrophage scavenging receptor SIRPα, is often expressed on cancer cells, enabling evasion of cellular removal in certain cancers. Similarly, CD47 is upregulated on cells within atherosclerotic plaques, and its inhibition has been shown to dampen plaque development by promoting clearance of apoptotic cells [102].

In animal models of atherosclerosis, CD47 blockade with magrolimab has been shown to reduce plaque burden and vascular inflammation [103]. Moreover, a retrospective analysis of patients on experimental therapy with magrolimab for non-Hodgkin’s lymphoma showed a potential reduction in vascular inflammation, reflected by decreased 18F-FDG uptake in carotid arteries [104,105].

In conclusion, while most approved ICIs targeting CTLA-4 and PD-1(L) pathways have been associated with pro-atherogenic effects and increased cardiovascular risk, magrolimab represents a promising exception. By modulating macrophage-mediated clearance mechanisms within atherosclerotic plaques, magrolimab may offer a novel therapeutic approach for the prevention or treatment of atherosclerosis. However, key constraints, including optimal timing of therapy relative to extent of atherosclerosis and the potential for destabilization of established plaques, require future translational investigation. Given the experimental nature of therapies such as magrolimab, as well as the exceedingly high current retail cost of immune checkpoint modulators, the therapeutic potential of this modality in atherosclerosis remains lofty, though experimental trials in high-risk patients may uncover populations that can benefit from such an expenditure.

### 3.3. Anti-Angiogenic Agents

Bevacizumab (anti-VEGF) and similar agents used in cancer therapy may reduce plaque neovascularization and associated hemorrhage. However, systemic inhibition of angiogenesis poses risks for wound healing and ischemic complications.

Angiogenesis is a fundamental process involved in both cancer progression and atherosclerosis. In cancer, neovascularization sustains tumor growth and facilitates metastasis, whereas in atherosclerosis, pathological angiogenesis contributes to plaque progression and destabilization. Given this mechanistic overlap, several anti-angiogenic cancer therapies known as VEGF signaling inhibitors (VSI) have been investigated for potential use in atherosclerosis.

#### 3.3.1. Bevacizumab (Anti-VEGF-A Monoclonal Antibody)

Bevacizumab is a humanized monoclonal antibody that binds to VEGF-A, blocking its interaction with VEGF receptors primarily expressed on endothelial cells, particularly VEGFR2 [106]. VEGF-A is a central mediator of angiogenesis and vascular homeostasis, and its expression is markedly elevated in advanced human coronary and carotid atherosclerotic plaques [107,108,109]. Bevacizumab is FDA-approved for several malignancies including colorectal cancer, non-small cell lung carcinoma, renal cell carcinoma, glioblastoma, and has even been used for cases of small bowel adenocarcinoma [110]. In these cancers, bevacizumab prolongs progression-free survival when combined with chemotherapy by disrupting tumor vascular supply [111]. Despite its clinical efficacy, bevacizumab is associated with cardiovascular toxicities such as hypertension, venous and arterial thromboembolism, proteinuria, and cardiomyopathy [62,112,113]. These adverse events are partially attributed to a reduction in nitric oxide and prostacyclin levels, accompanied by elevated endothelin-1, leading to endothelial dysfunction [62]. Hypertension occurs in 20–25% of patients, with a number needed to harm of six in phase III trials involving VSI [114,115].

The application of bevacizumab in atherosclerosis has yielded mixed preclinical results. In animal models such as mice on an atherogenic diet and rabbits with induced atherosclerosis, bevacizumab reduced intraplaque neovascularization and overall lesion size [116,117]. Furthermore, in ApoE3*Leiden mice, treatment with the VEGFR2-blocking antibody DC101 stabilized vein graft lesions by reducing size and intraplaque hemorrhage [118]. In contrast, systemic pan-VEGF inhibition in high-cholesterol diet ApoE−/− mice worsened lesion burden without altering plaque vulnerability [119]. Given these inconsistent findings in preclinical models and the well-established cardiovascular risks observed in oncology patients, systemic bevacizumab is unlikely to be a suitable candidate for repurposing in the treatment of atherosclerosis, though local or genetic delivery of VEGFA-blocking agents may still warrant exploration [46].

#### 3.3.2. Tyrosine Kinase Inhibitors with Anti-VEGF Activity

Several TKIs, including sorafenib, inhibit VEGF receptors in addition to other kinases involved in angiogenesis and cell proliferation. These agents are commonly used in renal cell carcinoma, thyroid cancers, and gastrointestinal stromal tumors, where they improve survival and reduce tumor burden, particularly in angiogenesis-dependent tumors [120].

A meta-analysis reported no significant difference in the cardiovascular toxicity profiles between the earlier mentioned direct VEGF-inhibitors such as bevacizumab and VEGF-targeting TKIs [115]. These toxicities arise from disrupted endothelial signaling and often mimic the clinical presentation of preeclampsia [121]. Both pazopanib and sunitinib have been associated with decreased cardiac function, while case reports have described cabozantinib and lenvatinib as inducing progression to end-stage renal disease [122,123].

Axitinib, a potent and selective VEGFR1–3 inhibitor with an acceptable safety profile in renal cell carcinoma, has shown promising plaque-stabilizing effects. In ApoE−/− Fbn1C1039G+/− mice with unstable plaques, axitinib reduced intraplaque neovascularization and hemorrhage and lowered coronary plaque burden. It also reduced myocardial infarction incidence [124]. Intraplaque angiogenesis is a key driver of atherosclerotic progression and destabilization, underlining the relevance of these findings [125,126]. These results suggest that axitinib may serve as an adjunct to statin therapy to prevent acute cardiovascular events such as myocardial infarction, stroke, and sudden cardiac death [53]. However, clinical data remain limited. In the AXIS phase III trial for metastatic renal cell carcinoma, grade 3 treatment-related hypertension occurred more frequently with axitinib than with sorafenib, prompting the recommendation of strict blood pressure monitoring during therapy [127,128].

Although preclinical studies highlight axitinib’s potential to stabilize atherosclerotic plaques, its clinical applicability remains uncertain due to the cardiovascular risks observed in oncologic settings. Nevertheless, careful selection of patients with neovascularization-prone unstable plaques and strict blood pressure control could help define a therapeutic niche for axitinib.

### 3.4. mTOR Inhibitors

The mammalian target of rapamycin (mTOR) is a protein kinase central to cell proliferation, immune regulation, and metabolic homeostasis. It acts via two distinct complexes, mTORC1 and mTORC2, which govern cell growth, cell survival and metabolic processes [129]. Rapamycin and its analogs (rapalogs), such as sirolimus and everolimus, selectively inhibit mTORC1 [130]. These agents are well established in oncology, where they are approved for malignancies including renal cell carcinoma, breast cancer, and pancreatic neuroendocrine tumors. Moreover, they are being used as immunosuppressive agents in solid organ transplantations [131].

The therapeutic effects of mTOR inhibitors extend to vascular pathology. Notably, sirolimus and everolimus are incorporated into drug-eluting coronary stents, where they prevent neointimal proliferation and restenosis [132,133]. This local vascular benefit prompted investigation into their systemic anti-atherosclerotic potential. In preclinical models, mTORC1 inhibition consistently attenuates atherosclerotic lesion development, with mechanisms including reduced SMC proliferation, impaired plaque monocyte recruitment and macrophage accumulation, reduced lipid accumulation in macrophages and SMC, and inhibition of HIF-1α-mediated neovascularization [134,135,136,137,138,139,140,141]. Rapamycin-treated ApoE−/− mice show up to 40% less cholesterol accumulation in the aortic arch [142]. One murine study even demonstrated improved cardiac function and survival in mice with advanced atherosclerosis following everolimus treatment [143]. However, another mouse study found no significant effect of everolimus on pre-existing lesions, suggesting reduced efficacy in advanced atherosclerosis [139].

In transplant patients, a population with elevated cardiovascular risk, the use of mTOR inhibitors offers real-world insights. Heart transplant recipients treated with everolimus exhibit reduced progression of cardiac allograft vasculopathy (CAV), characterized by decreased intimal thickening and fewer adverse cardiac events [144,145]. Similar signals have been observed in pulse wave velocity studies in kidney transplant patients, where sirolimus use correlated with lower arterial stiffness, a surrogate of atherosclerotic burden [146,147]. Despite frequent dyslipidemia in this setting, cardiovascular mortality does not appear to be elevated [144,148]. Interpretation of these outcomes remains limited by possible selection bias excluding patients with severe lipid abnormalities together with incomplete lipid-lowering therapy documentation across studies [149].

To be sure, rapalogs are known to induce cardiometabolic side effects, notably hypercholesterolemia, hypertriglyceridemia, and hyperglycemia [62]. Although common, these effects may be dose-dependent and manageable. Statins and metformin, which modulate the mTOR pathway through AMPK activation, may reduce these toxicities while enhancing anti-atherosclerotic effects. Supporting this, a murine study in ApoE−/− mice with CKD demonstrated that co-treatment with rapamycin and atorvastatin reduced atherosclerosis and improved lipid profiles more effectively than either agent alone [150].

Interestingly, recent advances in nanotherapy have further refined mTOR-based approaches. Rapamycin-loaded nanoparticles targeting inflamed plaques or lesional macrophages have shown promising results in ApoE−/− mice, leading to reduced inflammation and plaque burden with improved safety [151,152,153,154,155,156].

Taken together, mTOR inhibitors act on key pathogenic mechanisms of atherosclerosis. While systemic use is limited by metabolic side effects, data from transplantation and targeted delivery studies provide a solid foundation for their repurposing. Carefully designed trials, particularly those controlling for lipid-lowering therapy, are warranted to explore their full cardiovascular potential.

### 3.5. Emerging Therapies

Beyond the major oncological drug classes previously discussed, several other cancer therapies demonstrate preliminary potential for repurposing in the context of atherosclerosis. One example is the poly(ADP-ribose) polymerase (PARP) inhibitor niraparib, which is currently approved for certain indications in ovarian and primary peritoneal cancer [157,158]. Niraparib functions by selectively inhibiting PARP1 and PARP2, thereby disrupting DNA repair mechanisms in cancer cells [159]. In a recent study, niraparib in mice attenuated atherosclerotic progression and induced regression of established lesions, suggesting both preventive and therapeutic potential [10].

Another agent of interest is all-trans retinoic acid (atRA), an active metabolite of vitamin A, which regulates cell proliferation, apoptosis, and differentiation, and plays a key role in embryogenesis [160]. atRA is currently used in the treatment of acute promyelocytic leukemia [161]. Multiple preclinical studies have shown that atRA reduces atherosclerotic burden in both mouse and rabbit models. In ApoE−/− mice, atRA treatment led to significant plaque reduction, while a synthetic retinoid (potassium retinoate), combining atRA with 9-cis retinoic acid, demonstrated superior efficacy compared to clopidogrel and atorvastatin [162,163]. In addition, macrophage-specific deletion of the retinoic acid receptor α resulted in increased lipid accumulation and inflammation, supporting a regulatory role of atRA in cholesterol efflux and immune modulation [164]. Rabbit studies further showed that oral atRA reduced plaque size, enhanced endothelial function through endothelial NO activation, and decreased endothelin-1 levels [165,166,167,168]. Despite these benefits, rare but clinically relevant adverse effects have been reported, including hypercalcemia, acute pancreatitis, male infertility, thromboembolic events, and dermatological complications [169].

Although still in the early stages of investigation, agents such as PARP inhibitors and retinoids may be promising in the treatment of atherosclerosis. Further translational studies are warranted to evaluate their safety and efficacy in cardiovascular contexts.

## 4. Future Trials and Translational Directions

We believe that repurposing oncologic agents for atherosclerotic disease offers several potential benefits. First, it utilizes the vast body of mechanistic and clinical research already established in oncology. Second, it opens the door to novel therapeutic targets not traditionally explored in vascular medicine and surgery and expands options for patients who are neither surgical nor endovascular candidates. Third, it reflects a growing recognition of shared pathophysiological networks across diseases once thought to be distinct.

Overall, there are many opportunities for translation and application of oncologic drugs to atherosclerosis, as summarized in Table 1. However, significant translational challenges remain, including drug toxicity, long-term safety profiles, and the need for precise patient stratification. While preclinical data are promising, rigorous further evaluation is needed for safety, efficacy, and mechanistic rationale when repurposing oncologic agents in vascular populations. Notably, certain anti-cancer therapies that share antiatherogenic activity have also been associated with increased vascular complications, including accelerated atherosclerosis, hypertension, and thromboembolic events. The risk of exacerbating vascular complications must be properly weighed against the benefits that these potent oncologic agents may theoretically provide. Thus, despite shared biological pathways, it is critical to directly investigate the complex interplay between these agents and atherosclerosis through well-designed studies spanning preclinical, translational, and clinical domains.

Prospective cohort studies in cancer patients receiving oncologic therapies are a valuable approach for tracking atherosclerosis progression in real time. Serial imaging modalities, such as coronary CT angiography, carotid ultrasound, ankle-brachial indices, and 18F-FDG PET/CT, can detect and monitor subclinical atherosclerosis [170]. Additionally, more interventional modalities, such as intravascular ultrasound (IVUS) can generate real-time tomographic assessment of diseased vessels [171]. When combined with biomarker surveillance, these studies provide insight into the inflammatory and metabolic activity of vascular lesions. Circulating markers such as IL-6, myeloperoxidase (MPO), MMP-9, hsCRP, CD47, and LDL cholesterol have demonstrated utility in characterizing chronic vascular inflammation. These markers have also been expressed within cancer and serve as targets for oncologic therapies [172]. For example, IL-6 blockade has been extensively studied in oncology and is now being explored in cardiovascular prevention, while inhibition of CD47, a “don’t eat me” signal exploited by tumors, has shown potential to enhance clearance of apoptotic and necrotic material in atherosclerotic lesions [173]. Additionally, Midkine is another emerging mitogenic growth factor expressed in both cancer and atherosclerotic lesions across all disease stages that may be particularly relevant in older patients. While a multitude of cancer biomarkers exist, notable overlapping markers with atherosclerosis present a unique opportunity: by leveraging these, we can simultaneously improve risk stratification in atherosclerosis and repurpose existing oncologic therapies toward vascular applications [174].

Preclinical studies using atherosclerosis-prone mouse models and conditional knockout systems are also important in determining how individual oncologic drug classes modulate plaque formation, immune cell infiltration, and metabolic reprogramming. For example, targeting specific pathways such as PD-L1 (immune modulation), mTOR (cell proliferation), or VEGF (angiogenesis) can help with understanding both the therapeutic potential and vascular risks associated with each class.

To translate these findings into clinical practice, early-phase interventional trials should focus on high-risk cardiovascular patients with imaging- or biomarker-defined subclinical disease. Testing low-dose regimens of agents such as imatinib or rapamycin, drugs with more favorable cardiovascular safety profiles, can help determine whether these therapies reduce vascular inflammation or stabilize high-risk plaques. Quantification of disease progression would include advanced imaging and biomarker endpoints for early signal detection, mechanistic insight, and risk stratification.

Given concerns about systemic toxicity associated with anti-cancer drugs, advanced drug delivery systems represent a potential therapeutic strategy that avoids unnecessary systemic side effects [175,176]. Nanoparticle-based platforms that allow for site-specific delivery of oncologic agents, such as rapamycin-loaded nanoparticles targeting lesional macrophages, have shown potent anti-atherosclerotic effects in murine models [175]. Future trials should investigate the clinical utility of these technologies, particularly in patients with existing life-threatening comorbidities where localized inflammation is elevated and targeted intervention may yield the greatest benefit.

Overall, these multi-level investigations will be essential to disentangling the dual roles of oncologic agents as both vascular therapeutics and potential sources of cardiovascular/vascular toxicity. Such work will ultimately guide the safe and targeted integration of oncology-derived strategies into the prevention and treatment of atherosclerotic cardiovascular disease.

## 5. Conclusions

The exploration of oncologic agents as potential modulators and a novel treatment modality of atherosclerosis is largely due to a number of shared cellular behaviors. Dysregulated proliferation, chronic inflammation, neovascularization, and evasion of apoptosis all underscore the biological plausibility of cross-applying cancer therapeutics to vascular disease.

Emerging research increasingly supports the concept that atherosclerosis, particularly in its progressive stages, mirrors features of a smooth-muscle-tumor-like state. Clonal expansion of vascular smooth muscle cells (VSMCs), phenotypic switching to synthetic or osteogenic subtypes, and unchecked proliferative signaling mediated by pathways like PDGF, mTOR, and PI3K/AKT closely resemble tumorigenic processes. Recent lineage tracing and single-cell transcriptomic studies have demonstrated that plaques are often dominated by monoclonal or oligoclonal populations of VSMCs, analogous to early neoplastic transformation [8]. This paradigm shift not only deepens our understanding of atherosclerotic biology but also reinforces the rationale for applying anti-proliferative and metabolic-targeted cancer therapies in the vascular field. Recognizing atherosclerosis as a form of vascular pseudo-neoplasia may open entirely new avenues for therapeutic innovation.

Despite the strong mechanistic rationale, a central challenge to oncologic therapeutics is the toxicity profile of many agents. While agents like imatinib and rapamycin show favorable vascular effects with limited vascular toxicity in preclinical studies, others, such as VEGF inhibitors or certain second-generation TKIs carry additional risks of hypertension, thrombosis, and endothelial dysfunction, which can paradoxically worsen atherosclerosis progression. These concerns necessitate precision in dosing, patient selection, and delivery strategies. Immune checkpoint inhibitors, while mechanistically attractive, may also paradoxically exacerbate plaque inflammation and destabilization if not appropriately monitored. Additionally, regulatory constraints may pose hurdles; the aforementioned anti-cancer agents were approved under oncology indications with careful consideration of risk-to-benefit ratios, whereas approval for vascular disease will require extensive long-term safety data in otherwise stable populations. Lastly, cost must be a consideration. Oncology drugs are very expensive; just the cost of imatinib is around $75,000–$100,000/year, and when used for off-label purposes, they may be even more expensive [177]. Finally, target selection remains nebulous. Unlike some tumors and well-researched cancers, atherosclerotic plaques are heterogeneous and dynamic, which may complicate the identification of molecular targets most likely to yield therapeutic benefit [178].

We must also contend with limited biomarkers and imaging tools specific to therapeutic response in atherosclerosis. Surrogate endpoints such as vascular PET imaging, serial coronary CTA, and inflammatory biomarkers must be further validated to support early-phase clinical trials. Finally, longitudinal cohort studies in cancer patients receiving oncologic therapies can offer real-world insights into vascular effects, both harmful and potentially beneficial.

Ultimately, reimagining atherosclerosis through the lens of oncology not only opens the door to novel therapeutic strategies but also continues to reinforce the constant innovation that is seen in vascular medicine and surgery. Advancing this translationally will require close collaboration among oncologists, vascular biologists and surgeons, cardiologists, and pharmacologists. We also are aware of the strong existing barriers that will require careful dose optimization, biomarker-driven patient selection, and potentially novel formulations or delivery strategies to minimize systemic toxicity while preserving efficacy. Collaborative efforts between vascular and oncologic trial frameworks may accelerate the design of early-phase translational studies in this space. By targeting the shared cellular and molecular machinery that drives both tumor growth and plaque progression, we believe we can gradually approach a future in which atherosclerosis can be managed at its root.

## Figures and Tables

**Table 1:** Classes of oncology drugs with applications for atherosclerosis.

Drug Class	Drug	Target	Current Indications	Cardiovascular Effects	Toxicities	Mechanism in Atherosclerois
Anti-Angiogenic Agents	Bevacizumab	VEGF-A	Colorectal, NSCLC, RCC, glioblastoma	Hypertension, thromboembolism	Proteinuria, cardiomyopathy	Inhibits angiogenesis, endothelial dysfunction
Immune Checkpoint Inhibitors	Magrolimab	CD47	Non-Hodgkin’s lymphoma	Reduced vascular inflammation	Not specified	Enhances efferocytosis, plaque stabilization
PARP Inhibitors	Niraparib	PARP1, PARP2	Ovarian, peritoneal cancer	Anti-atherosclerotic in mice	Not specified	Reduces SMC hyperproliferation
Retinoids	All-trans retinoic acid (atRA)	Retinoic acid receptors	Acute promyelocytic leukemia	Plaque reduction, improved endothelial function	Hypercalcemia, pancreatitis, thrombosis	Regulates cholesterol efflux, reduces inflammation
Tyrosine Kinase Inhibitors (TKIs)	Imatinib	BCR-Abl	CML, Ph+ ALL	Anti-atherogenic, reduces cholesterol and plaque	Generally safe CV profile	Reduces inflammation, cholesterol, and oxidative stress
	Nilotinib	BCR-Abl	Imatinib-resistant CML	Pro-atherogenic, increased CV events	CAD, MI, stroke, PAOD	Pro-thrombotic, endothelial damage
	Ponatinib	BCR-Abl	Resistant CML/Ph+ ALL	High CV risk	Cardiac, cerebrovascular, peripheral arterial events	Limited data, suspected pro-thrombotic
	Dasatinib	BCR-Abl, multikinase	CML/Ph+ ALL	Atheroprotective in mice	Pulmonary HTN	Reduces oxLDL uptake, macrophage modulation
	Saracatinib	Src family kinases	Breast, lung cancer (investigational)	Atheroprotective	Not specified	Reduces plaque inflammation, neointima
	Sorafenib	VEGFR, multikinase	RCC, HCC	HTN, potential vascular toxicity	Hypertension, ischemia, QT prolongation	Reduces NO, increases resistance
	Axitinib	VEGFR1‚ Äì3	RCC	Plaque stabilizing in mice	Hypertension	Reduces neovascularization, plaque burden
mTOR Inhibitors	Rapamycin (Sirolimus)	mTORC1	Cancer, transplantation	Anti-atherogenic, used in stents	Dyslipidemia, hyperglycemia	Reduces SMC proliferation, lipid accumulation
	Everolimus	mTORC1	Cancer, transplantation	Reduced CAV, arterial stiffness	Similar to sirolimus	Inhibits proliferation, inflammation
